# Scanning Microscopy Techniques as an Assessment Tool of Materials and Interventions for the Protection of Built Cultural Heritage

**DOI:** 10.1155/2019/5376214

**Published:** 2019-02-26

**Authors:** Antonia Moropoulou, Elisabetta Zendri, Pilar Ortiz, Ekaterini T. Delegou, Ioanna Ntoutsi, Eleonora Balliana, Javier Becerra, Rocío Ortiz

**Affiliations:** ^1^Laboratory of Materials Science & Engineering, School of Chemical Engineering, National Technical University of Athens, 9 Iroon Polytechniou Str., Zografou Campus, Athens 15780, Greece; ^2^Department of Environmental Sciences, Informatics and Statistics, Ca' Foscari University of Venice, Mestre, (Venice), Italy; ^3^Departamento de Sistemas Físicos, Químicos y Naturales, Universidad Pablo de Olavide, ES-41013 Seville, Spain

## Abstract

Scanning microscopy techniques have emerged as powerful scientific tools for analysing materials of architectural or archaeological interest, since the commercialization of the first scanning electron microscopy instrumentation in the early 60s. This study is aimed at reviewing and highlighting the significance of several scanning microscopy techniques employed in the protection of built heritage. The diffusion of scanning electron microscopy with energy-dispersive X-ray spectroscopy analysis (SEM-EDX) is proven to be the widest among the available scanning microscopy techniques, while transmission electron microscopy (TEM) applications are steadily present in the field of built heritage protection. The building material characterization, the weathering mechanism investigation, and the development of compatible and performing conservation materials are some major research areas where the application of the aforementioned techniques is discussed. The range of techniques, along with aspects of instrumentation and sample preparation are, also, considered.

## 1. Introduction

### 1.1. Materials Science in Built Cultural Heritage

A vast variety of building materials has been used for constructive or artistic purposes since prehistory: natural stones, earth materials, bricks, lime, and gypsum mortars prevail in the history of architecture [1]. From the mud constructions in Ancient Egypt and the marble post and beam form of Ancient Greek Temples, to the Roman “concrete” infrastructure and the gypsum plasters of Arabic architecture, the interface lies on the craftsmanship regarding the use of materials and the evolution of techniques [2, 3]. The domination of Portland cement in architecture since the early 20th century inferred the decrease in the usage of natural building materials. Nevertheless, the multilateral values' appreciation of historic structures never ceased.

Nowadays, the concept of cultural heritage preservation includes cement or modern concrete constructions, as well. In parallel, the tendency for sustainable and alternative architecture has turned the interest to the use of traditional building materials in modern constructions. The adverse environmental impact, escalated by the climatic change and anthropogenic activities, dictates the need to increase the scientific knowledge on the behaviour of both traditional-historic and contemporary building materials, in order to provide solutions. Conventional and new intervention materials and methods need to be evaluated and monitored in terms of effectiveness, reversibility (whenever applied), and compatibility. Within the above framework, the use of natural and chemical analytical methods in the field of architecture and artworks conservation, as well as in humanities, has been established as essential for unveiling the evolution of materials and techniques used by mankind throughout geographical regions and different time periods. Historic structures and their comprising materials are subjected to constant research in order to understand their behaviour and properties. This approach has, also, emerged as a necessary prerequisite in order to plan appropriate restoration and conservation strategies for the rehabilitation of historic and modern constructions.

Regarding the characterization of historic materials, the analytical methodology that needs to be adopted depends on the questions that are to be answered and on the amount of sample available. The approach is differentiated per case; for example, a sample that derives from an archaeological site sets chronological and provenance issues as the primary to be addressed, while when it comes to the restoration of a historic structure, the knowledge of the authentic building materials is the base for designing compatible conservation interventions and their durability assessment.

In addition, the knowledge of the technological development of building materials such as limes, concretes, mortars, metals, tiles, and glasses is of high anthropologic and historical interest, since it sheds light to the development of civilization and commerce throughout centuries, while pigments, binders, or additives identified in mortars or paintings allow the in-depth investigation of the utilized traditional and local techniques, contributing to the record of their progress.

### 1.2. An Overview of the Techniques Used in the Fields of Building Materials and Cultural Heritage

While many characterization techniques have been practised in the field of building materials, new techniques and methodologies are constantly emerging. Chemical analyses like atomic absorption spectroscopy/atomic emission spectroscopy (AAS/AES), X-ray fluorescence (XRF), inductively coupled plasma optical emission spectrometry (ICP-OES), gas chromatography (GC), mass spectrometry (MS), laser-induced breakdown spectroscopy (LIBS), high-performance liquid chromatography (HPLC), laser-induced fluorescence (LIF), particle-induced X-ray emission (PIXE), and particle-induced gamma-ray emission (PIGE) are used. However, they present significant limitations, as they are not able to detect the exact composition and structure of materials, they require a great amount of sample or complicated preparation routes, and they are time-consuming.

Fourier transform infrared (FTIR) and Raman spectroscopic techniques offer the chemical characterization of small size samples, while X-ray diffraction (XRD) analysis provides the mineralogical composition of materials under examination, but all of them lack the ability to offer morphological information.

Each one of the abovementioned analytical techniques may be employed in order to address special issues of cultural heritage like the identification of pigments and binding media [4, 5], in artworks or the analysis of artefacts like historic textiles [6] and metallic objects [7]. The advent of in situ instrumentation, e.g., in situ XRD, XRF, LIBS, and Raman, has revolutionized the application efficacy of these techniques on monuments, since sampling in the field of cultural heritage is rather restricted, and the mass of each finally acquired sample is limited to the minimum. Nevertheless, the detection limit of these in situ performing techniques is lower compared to the respective lab instrumentation ones, while the geometry of the in situ equipment is not always practical or functional for demanding applications in the field of built heritage, mainly due to accessibility reasons (scaffolding, concave- or convex-shaped structures, etc).  Table 1 summarizes some of the major analytical techniques used in the field of architectural heritage and building materials science, presenting the type of information acquired by their utilisation, the sample requirements, and their major limitations.

### 1.3. Scanning Microscopy Techniques in the Field of Built Cultural Heritage

In the field of building materials characterization and especially in the field of architectural heritage, knowing at microscopic level the composition, texture, and structure of building materials has been a revolution. The classical approach in microscopy involves the use of optical and petrographic microscopy. These techniques are relatively simple and versatile tools for the textural examination and the petrographical/mineralogical composition of a range of building materials, such us stones, limes, mortars, tiles, bricks, glasses, metals, and wood. Confocal optical microscopy has, also, emerged as an in-depth morphometric tool and offers the possibility for the 3D reconstruction of the sample surface [8].

The use of scanning microscopy techniques in the examination of historic building materials has evolved as a well-established analytical tool soon after the commercialization of the first scanning electron microscopy instrument (SEM) in the early 60s [9]. SEM coupled with energy-dispersive X-ray spectroscopy (EDX) has emerged as a fundamental technique for the examination of building materials, since it allows the investigation of the surface microstructure in the desired detail and offers the chemical composition of the examined spot or area. This spatially defined chemical and morphological information of the sample under examination offers valuable information about the components, the manufacture technology, the degradation mechanisms, and others.

The main advantages of the SEM-EDX analysis are

1. the lack of need for preliminary preparation and thus the nondestructive treatment of valuable specimens
2. the nanoscale resolution offered by modern SEM and thus the precision level
3. the variation of the examined region (from several tens of centimetres to nanometres) by adjusting the magnification
4. the qualitative and quantitative analysis offered by coupling SEM with EDX microanalysis

Transmission electron microscopy (TEM) has, also, been introduced in the field of cultural heritage materials; the information in this case is, as well, both morphological and chemical (when combined with EDX), and the magnification level here is even to subnanometre scale. However, the small size of the analyzed area (only a few square micrometres) does not permit a broad sample examination, while sample homogeneity is required for a full-potential TEM analysis.

Furthermore, another scanning microscopy technique, recently developed, is the atomic force microscopy (AFM). It was commercially introduced in 1989 and is a nanoscale technique aiming at measuring the topography of a nonconductive sample, providing 2D and 3D images. There is some work published using this technique in the field of cultural heritage, e.g., [10]. However, this technique has a limited application in cultural heritage samples, since their surface texture and roughness is above nanoscale in most of the examined cases. Nevertheless, this technique is increasingly gaining ground in the assessment methodology of conservation interventions that involve nanomaterial applications [11–14]. In Table 2, the main advantages and limitations of SEM, TEM, and AFM techniques in the field of build cultural heritage are summarized.

### 1.4. The Diffusion of SEM-TEM Techniques in the Built Cultural Heritage Research Field: A “Numeric” Evaluation by Scopus and Google Scholar

As already discussed, scanning and transmission electron microscope techniques have been widely applied in the characterization and in the evaluation of the performance of new and traditional methods for the preservation of monuments. In an effort to numerically evaluate how really diffused SEM and TEM techniques are in the field of architectural heritage, a bibliographic research was conducted in two of the most common search databases: Scopus (https://www.elsevier.com/solutions/scopus) and Google Scholar (https://scholar.google.com). Hereby, the results obtained are presented and discussed.

The publications of these platforms derive from different sources, and in particular Google Scholar provides data related to a larger number of resources not limited to the traditional academic research field. Scopus, indicated as the largest abstract and citation database of peer-reviewed literature, counts about 18500 peer-reviewed journals from over 5000 publishers, 1800 open access journals, 250 conference proceedings (for a total of 4.8 million documents), 425 commercial publications, 350 book series, Medline (full coverage), 375 million quality web pages through Scirus 17, and 24.8 million patent registrations. As stated in the guidelines, Google Scholar allows you to search through many disciplines and sources: documents approved for publication, theses, books, abstracts and articles of academic publishers, professional orders, databases of studies not yet published, universities, and other academic organizations. However, Google Scholar does not allow a selection of materials, so you only get information about the number of publications related to a given topic.

The comparison among these two search engines can give some really interesting conclusions in particular on how different results can be obtained starting from the same key words. The key words used in the search were “scanning electron microscopy” AND “cultural heritage” or “transmission electron microscopy” AND “cultural heritage.” The period of time considered was between 2005 and 2018. Using the key words “scanning electron microscope” AND “cultural heritage,” the research on Scopus results in 200 documents, 20 of which as open access. The majority of these are articles in journal (82.5%), papers related to conferences (13.5%), and in minor number reviews (4%). All the documents are spread in a total number of 110 journals, including journal proceedings. Among these, the Journal of Cultural Heritage is the one with the higher number of papers on SEM applications (10 papers), followed by the Journal of the Total Environment (9 papers), Applied Physics A Materials Science and Processing (8 papers), Surface and Interface Analysis (7), and Applied Surface Science (6). The most cited paper (71 citations) focused on the deterioration due to fungal and bacterial growth of marble treated with a synthetic resin [15]. The results of the research in Google Scholar are quite different and very interesting. Using the same key words, we obtain 7650 records from 2005 to 2018.

The results obtained were analyzed using an external tool created by Anne-Wil Harzing (University of Melbourne), distributed free of charge on the web and called Publish or Perish (PoP) (http://www.harzing.com/pop.htm) which calculates, using Google Scholar data, citation indexes for individuals, magazines, and institutions. Applying again the key words “scanning electron microscopy” AND “cultural heritage,” PoP selects 780 publications between 2005 and 2018. The most cited paper (466 citations) is an archaeological study of 2010 published in Nature concerning the use of stone tool manufacture [16]. Among other articles, an interesting work concerning the study of historical mortars by Elsen (255 citations) is also reported [17], not present in the list obtained through the previous search in Scopus.

Another important paper (with 133 citations) is related to the identification of artist materials, where SEM is applied for comparing analytical results obtained by other in situ techniques [18]. As in the previous case, this article and other interesting papers are not reported in Scopus.

The research about the application of TEM in heritage science follows the same approach, using “transmission electron microscopy” AND “cultural heritage” in Scopus and Google Scholar as key words in the years 2005-2018.

In Scopus, 21 documents were pointed out published in 19 journals, among which are 27.8% in the subject area of Materials Science, 18.5% in Chemistry, 18.5% in Physics and Astronomy, 11.1% in Engineering, 5.6% in Arts and Humanities, and 5.6% in Computer Science. The 13% of the remaining documents are spread out in other subject areas. The majority of these documents are articles in journal (71.4%), paper conferences (14.3%), reviews (9.5%), and book chapters (4,5%). Again as for SEM, the journal with the highest number of papers in which TEM is cited is the Journal of Cultural Heritage (3 papers) and the only three papers found are the same resulting for the SEM applications. One of the most cited papers (69 citations) is a study on the carbonation process of nanolime, used as consolidant in cultural heritage [19].

**Figure 1:**
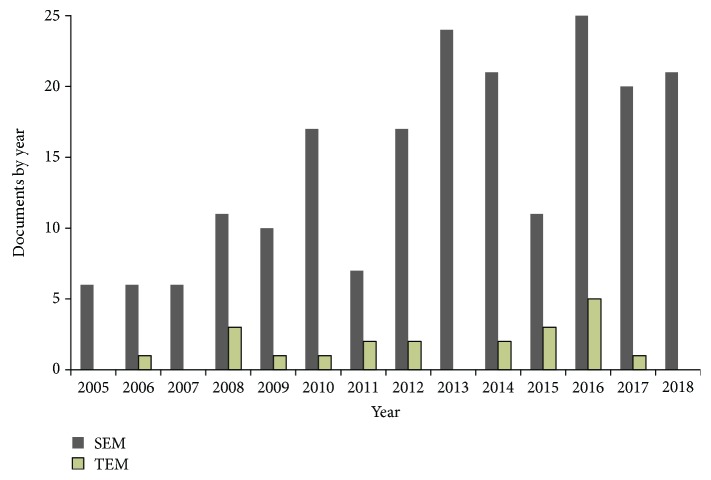
Diffusion over the time of the papers that report SEM/TEM application in cultural heritage studies.

In Figure 1, the distribution of the papers related to SEM and TEM applications per year is reported, on the base of data coming from Scopus. The application of SEM analyses in the field of Cultural Heritage has increased over time and seems to have reached a “threshold,” probably due to the transition from “technique for the research field” to “routine technique.” TEM is much less widespread, probably due to the cost of the instrumentation, the difficulty in preparing the samples, and its reduced applicability. For these reasons, the trend of the application of the TEM technique in cultural heritage does not show a regular increase over time.

These considerations refer to a significant number of publications, which only partially reflect the actual situation, since not all the journals are included in Scopus.

In Google Scholar, 1900 papers are picked but as previously the data are not analyzed. For this reason, the same key words were selected via PoP and 997 publications were highlighted in the years 2005-2018. The most cited (753 citations) is a book in which a paper about the application of gels for cleaning artworks materials is presented [20]. The articles picked by POP are speared in almost 450 different journals, proceedings, and websites.

Based on this short research, it is evident that the selection of the key words and the database can give very divergent results. Moreover, the use of simple and straightforward key words, as SEM-CH and TEM-CH, used to facilitate the search does not give complete data. In some cases, using additional words or just changing the combination of the words, we found papers—not included in the Scopus and Google searches—in which SEM and TEM techniques are used. Based on the results, while TEM is mainly used for the development and applications of nanotechnology, SEM can be considered as a common analytical technique in all areas of cultural heritage research, starting from the traditional material characterization to the development of innovative and breakthrough methodologies.

## 2. SEM-EDX Analysis

### 2.1. Instrumentation and Sampling Preparation

The technique is based on an electron beam scanning the sample in parallel lines in order to obtain an image at the required level of zoom. The electron beam is produced in a high-vacuum column, where the heating of a filament (tungsten, lanthanum hexafluorine, etc.) generates the electrons. These electrons are then accelerated by a potential difference controlled by the operator, which may vary between 200 V and 500 kV, depending on the sample.

There are different types of columns, magnetic lenses, and overtures which are used to achieve the focalization and condensation of the originally disperse electron beam. At the end of the column, a magnetic device is responsible for the scanning movement of the beam over the surface of the sample.

In the conventional electron microscopes, the chamber is also subject to high vacuum (approx. 10^−7^ atm) to avoid the beam and the different signals produced by the interactions colliding with the gases that it may contain. The new-generation SEM, called environmental (ESEM) can also work at low pressures to examine samples that cannot resist such high pressures, such as biological samples.

One of the many advantages of the technique in cultural heritage is the versatility of the detectors that can be coupled to it to study the interactions generated by different kinds of signals: secondary electrons, backscattered electrons, Auger electrons, X-rays, cathodoluminescense, etc. The detectors most used in cultural heritage are (i) the secondary electron (SE) detector, which renders topographical images as a consequence of the collision of the primary electron beam, (ii) backscattered electron detector (BSE), which renders the so-called compositional images, since elements of higher atomic number are displaying by higher contrast, and (iii) energy-dispersive X-ray analysis which makes a spectrum of X-rays emitted by the specimen on the basis of their energy. These different phenomena can take place over the sample simultaneously while it is being observed, making this fact the greatest advantage of SEM-EDX technique.

The use of accelerated electrons as a lighting source instead of visible light has improved the magnification achieved. Moreover, its depth of field allows it to focus at different heights, which makes the three-dimensional images obtained quite realistic. Because of this feature, samples observed with a scanning electron microscope do not have to be flat, as in the case of optical microscopy. Images obtained are colored in different levels of greyscale corresponding to diverse information depending on the detector used.

At the beginning of SEM-EDX applications, a sample under investigation required special treatment which included the application of gold and/or carbon as coatings on its surface for increasing its conductivity. Application of gold resulted in SEM images of improved quality, where application of carbon resulted in more accurate EDX analysis. However, sample treatment nowadays is not required, due to the advances of ESEM-EDX.

In cultural heritage, the scanning electron microscope is usually used coupled to an EDX microanalyzer, which allows chemical elemental analysis of the observed spots or areas to be conducted. SEM-EDX is one of the most interesting techniques used in the study of material characterization and decay diagnosis in architectural heritage, mainly because of its ability to obtain topographical, morphological, and chemical information with little to no sampling preparation, as previously discussed. New software tools related to microanalysis revolutionized its application, since elements' distribution on the examined area can be depicted on the respective SEM images through scan lines or surface maps using pseudo-colors.

In addition, further research has flourished in the thematic area of digital processing of SEM images in an attempt to quantify the qualitative topographical and morphological results that are acquired by SEM. This research field is focused on the porosity estimation of building materials [21, 22], as well as in the assessment of conservation interventions [23].

### 2.2. SEM-EDX Applied in the Protection of Built Cultural Heritage

The adverse effect of environmental loads on construction materials is manifested through the various degradation processes detected on both modern and historical buildings. The type and extent of each decay pattern may vary according to the examined building material, the specific environmental factors, the microclimate of the construction, anthropogenic activities (vandalism), abandonment, etc. Both historical and modern constructions need conservation/restoration or repair interventions. The research over the most appropriate intervention methodologies and on the development of new materials is vast due to the challenges posed by environmental and socioeconomic factors. The turn to scientific approaches regarding the protection of built cultural heritage and the trend of designing more sustainable materials and architectural forms have enriched the research to a great extent.

#### 2.2.1. Characterization and Decay Diagnosis of Stone

In urban environments, the development of superficial crusts, the acid attack, and the accumulation of pollutants are to be faced. These procedures should be well documented in order to reveal the exact degradation mechanism and in order to design the next steps for eliminating their negative effect. SEM-EDX analysis has been widely used within this purpose. The sulphation of calcareous stones is a common degradation process in urban environments. Th. Skoulikidis pioneered in the utilisation of SEM for the examination of gypsum formation on ancient marbles, suggesting a sulphation mechanism based on the electrochemical cell theory [24]. Since then, SEM has been used for the observation of gypsum formation on various calcite lithotypes during which it was proven that the lateral distribution of gypsum crystals depended on the mineralogical composition [25].

The formation of black crusts on architectural surfaces made of calcareous stones has been investigated through SEM-EDX analysis at several important European monuments such as the Acropolis monuments in Athens, Greece [24, 26] and the Cathedral of Seville in Spain [27], among many others. The SEM-EDX analysis of a series of samples collected from buildings in Lisbon, Portugal, constructed from various traditional and modern building materials revealed the presence of hazardous pollutants and demonstrated the role of different factors in the formation of the crusts [28]. Furthermore, the investigation of several crusts on the ancient marbles of the Sanctuary of Demeter in the industrial atmosphere of Eleusis in Greece, using electron probe microanalysis (EPMA) and SEM, resulted in the in-depth compositional analysis of the samples, classifying the formulation mechanisms of the identified crusts [29].

**Figure 2:**
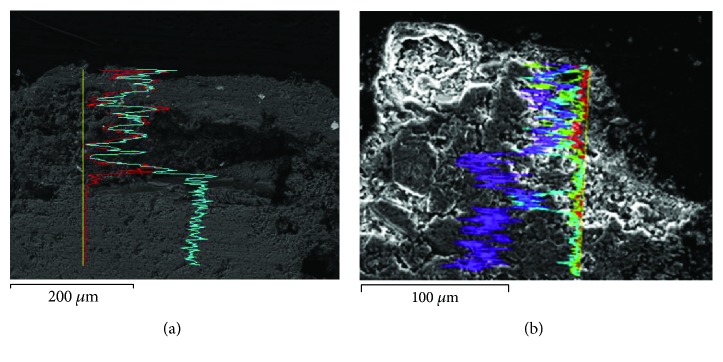
SEM-EDX analysis of a black crust over limestones from façades of XIII-XV centuries from Seville (Spain). (a) The blue EDX spectrum is calcium, and the red EDX spectrum is sulphur; (b) the purple EDX spectrum is calcium, and the turquoise EDX spectrum is sulphur. EDX shows the presence of a compound of sulphur and calcium on the surface (gypsum) and the matrix of calcium from limestone.

The SEM images (Figures 2(a) and 2(b)) show two samples of black crust perpendicular to the surface. EDX spectra of Figure 1(a) show the content of calcium (blue EDX spectrum) and sulphur (red EDX spectrum), while EDX spectra of Figure 2(b) show the content of calcium (purple EDX spectrum) and sulphur (turquoise EDX spectrum) with aluminium spectrum (red line) and silicon spectrum (green line). The inner part is mainly composed of calcium, due to calcite of the limestone, while the surface is composed of sulphur and calcium, due to the gypsum generated as a reaction between SO_2_ from pollution and calcium carbonate from limestone. This layer captures other particles present in the atmosphere and produces a characteristic grey-black color. SEM-EDX allows analysing the process to assess the effect of pollution on built heritage and evidence the difference of texture and porosity in the new surface [30]. The line scan is a useful tool for surface analysis and layer characterization of weathering forms.

Another important advantage of SEM-EDX application in the investigation of crusts is the detection of any patina occurrence. Detection of patinas and polychromes is of high importance due to historical, physicochemical, and aesthetical reasons; and as such they have to be preserved during the application of conservation interventions.

During the decay diagnosis of the building materials of the main facade of the National Archaeological Museum of Athens, Greece (NAM), the characteristic decay pattern of black crust was identified at many of the marble surfaces under examination [31, 32].

**Figure 3:**
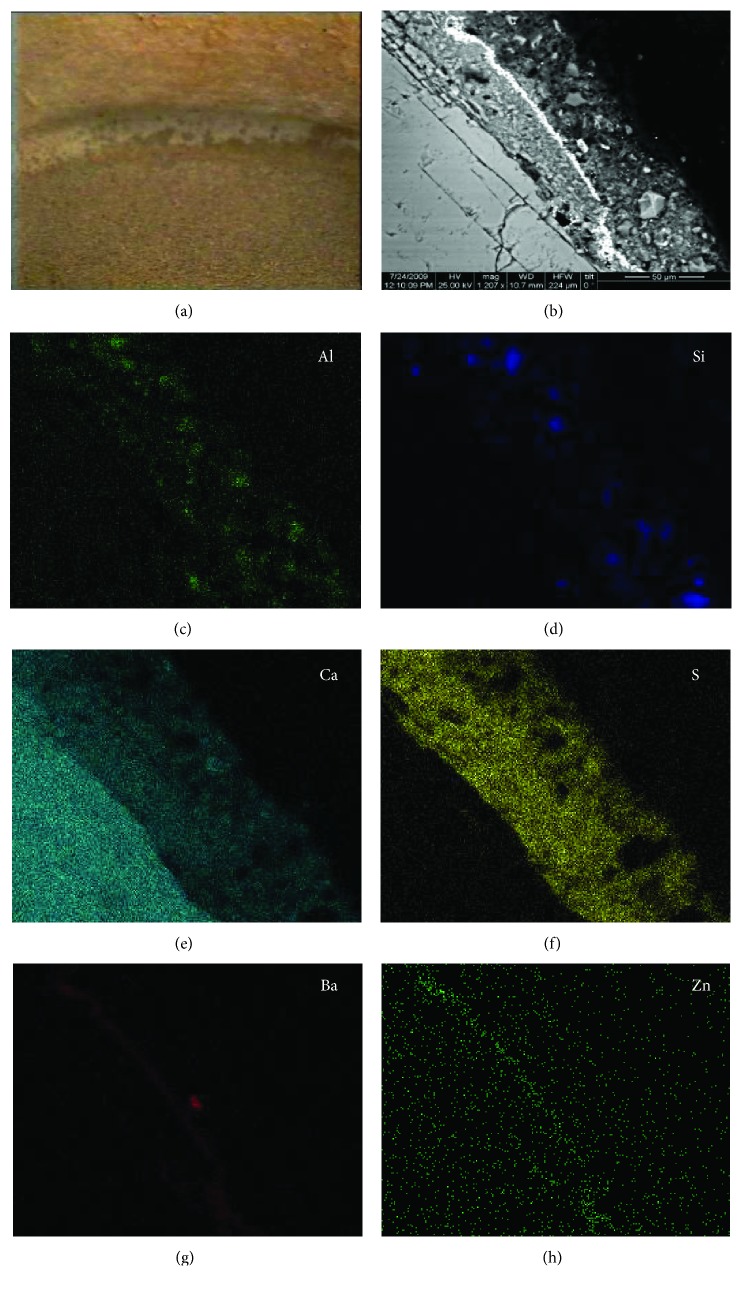
(a) Digital microscopy image of black crust on an outdoor marble capital of the National Archaeological Museum of Athens, Greece, (×25); (b) SEM BSE image of the black crust under examination; (c, d, e, f, g, h) EDX mapping of the detected elements, Al, Si, Ca, S, Ba, and Zn, respectively.

The cross sections of core samples taken from the marble capitals of the NAM were investigated by SEM and EDX mapping.  Figure 3 presents a representative image of a cohesive black crust constituting of (a) an inner, compact, and clear gypsum layer of microcrystalline texture, (b) an outer gypsum layer rich in Si and Al, and (c) a layer of low thickness, rich in Ba and Zn, located in between the two aforementioned gypsum zones and depicted as white line in the SEM BSE image (Figure 3(b)). The examined sample holds a typical stratification of a black crust that is a clear gypsum layer inwards and an external gypsum layer which contains aluminium silicate compounds and presents dark coloring, due to the accumulation of black depositions as the digital microscopy image demonstrates (Figure 3(a)). However, the Ba-Zn middle layer was not typically expected to be identified, and therefore studying its origin is of high importance, since if it is a patina, it should be recorded and preserved whenever conservation treatments are to be applied.

**Figure 4:**
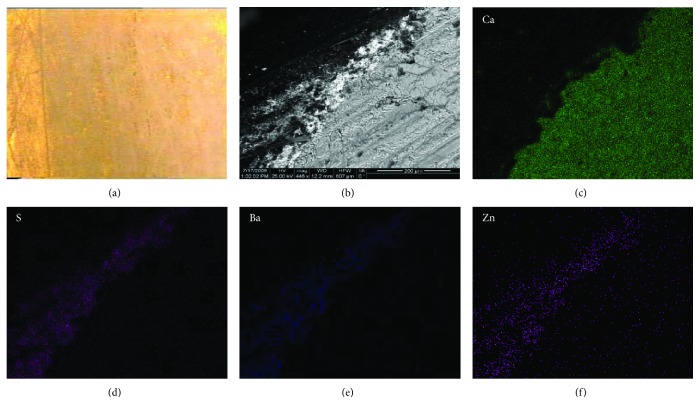
(a) Digital microscopy image of coloring decoration imitating gold on an indoor marble capital of the National Archaeological Museum of Athens, Greece (×25); SEM BSE image of the colored area under examination; (b, c, d, e, f) EDX mapping of the detected elements, Ca, S, Ba, and Zn, respectively.

Within this investigation framework, sampling, from corresponding areas of the indoor marble capitals of the entrance hall of the NAM, took place. The indoor marble capitals of NAM hold several coloring decoration residues of gold, blue, and red hue that can be visually observed. In addition, conservation treatments have never been performed on these architectural surfaces, according to the museum records. Therefore, whichever decoration technique had been used, it can be dated back to the initial construction of the historic building at the end of the 19th century.  Figure 4 displays representative results of the SEM and EDX mapping investigation of an indoor marble sample with a golden hue, due to the use of an organic pigment [32], cut in cross section.

Figures 4(b)–4(f) demonstrate the presence of a thick layer rich in Ba, Zn, and S, on the top of the marble surface and under the organic pigment layer (Figure 4(a)). Ιt is concluded that this layer is the white pigment of lithopone (main chemical composition: ZnS 28% and BaSO_4_ 72%), which was introduced at the second half of the 19th century, and it was also used as a preparation medium and base for several coloring decorations such as gold [33]. It is produced according to the coprecipitation reaction of barium sulfate and zinc sulphide, as shown below:

(1) $$\begin{array}{c} \text{BaS}+{\text{ZnSO}}_{4}\to \text{ZnS}+{\text{BaSO}}_{4}. \end{array}$$

Therefore, it can be concluded that the Ba-Zn layer identified in between the gypsum layers at the black crust sample of the outdoor capital of the NAM is a remnant of lithopone pigment. This is important historical evidence pointing out that the outdoor capitals of the NAM historic building were colored somewhere between the end of the 19th century and the beginning of the 20th century, the period that lithopone use was flourishing. Therefore, this lithopone layer is considered a patina and it has to be preserved whenever conservation interventions take place in the future at the historical building of NAM.

The salt-induced decay phenomena on natural stones are another important and severe decay factor that has to be addressed. Capillary rise and/or marine aerosols are the predominant sources of salts in stone structures. Stone microstructure properties, such as porosity and pore size distribution, along with environmental conditions affect salt crystallisation processes. Granular disintegration of stones, cracks, and even detachments of whole masonry parts can take place as a result of salt crystallisation within porous systems.

SEM-EDX analysis can be used for determining the morphology and the chemical composition of salt crystals, as well as for mapping the superficial and in-depth distribution of diverse saline phases in the building stones. Salt efflorescence and subefflorescence phenomena of the Falla Chapel's building stones at the Cathedral of Cadiz in Spain were documented regarding their spatial distribution on the monument, their seasonal variation in correlation to microclimate, the kind of salt detected due to the mixed urban and coastal environment, and the degree of deterioration induced by each one [34]. Moreover, SEM-EDX has been used to record and document different halite morphologies in the investigation of the effects of marine aerosol dry deposition on monuments of the Medieval City of Rhodes, Greece [35].

**Figure 5:**
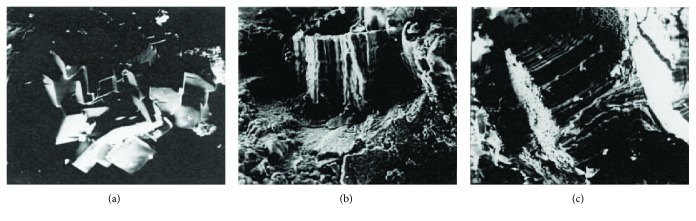
SEM images of different growth patterns of NaCl crystals in biocalcarenite porous stone, Medieval City of Rhodes, Greece. (a) Growth of NaCl isometric crystals (magnification ×160); (b, c) growth of NaCl columnar crystals (magnification ×200).

The Medieval City of Rhodes is an exceptional paradigm of marine environment, demonstrating how NaCl crystallisation acts on highly porous stones, as the biocalcarenite found at Rhodes monuments. The growing mechanism of NaCl crystals was monitored through the utilisation of SEM and EPMA, while their possible disruptive effect on pores' walls was documented in samples taken from historical masonries [36]. In this work, it was shown that well-shaped isometric crystals of NaCl are developed in stone pores, located at the lower and inner parts of the historical masonries examined, that is, areas where samples presented comparatively high humidity concentrations (Figure 5(a)). Thus, the growth of NaCl isometric crystals is favored in a pore, when the supply rate of salt solution is higher compared to the evaporation processes. In contrast, when the evaporation rate is higher in comparison to the salt solution supply, then columnar crystals of NaCl are developed in a pore (Figures 5(b) and 5(c)). This kind of condition was found at higher and external surfaces of the historical masonry studied, where low humidity values were measured at the samples under investigation.

Therefore, the above presented differences in the crystal growth patterns of NaCl designate the differences in the evaporation rates of the historical stone masonries under investigation both height-wise and depth-wise [36].

However, whatever the case of crystal growth pattern is, as NaCl crystals grow in the pores, disruption of the pore walls may occur, depending on the crystallisation pressure applied by the growing crystals against pore walls, microclimatic conditions, and stone properties.

Besides the inorganic types of decay described above, biologically induced damage is another important deterioration factor of stone. Biodeterioration, that is, degradation of stone surfaces by lichens, fungi, algae, and bacteria, is a process of physical, chemical, biological, and aesthetical nature [37]. The stone matrix is mechanically damaged by hypha penetration and thallus development, while chemical decomposition of stone takes place due to the excretion of organic acids, some of which induce color change on the stone surfaces [38], while the biological effect on stone texture results in mineral substitution or leaching [39].

Colonization of microorganisms on stone depends on environmental conditions as moisture, temperature, light exposure, and nutrition availability [40].

SEM application in biodeterioration studies is rather helpful in the identification and classification of the microorganisms evident on the historical substrates, based on their shape and morphology characteristics. Furthermore, information on the biodeterioration mechanisms and their impact on the stone texture can be figured out by SEM observations.

**Figure 6:**
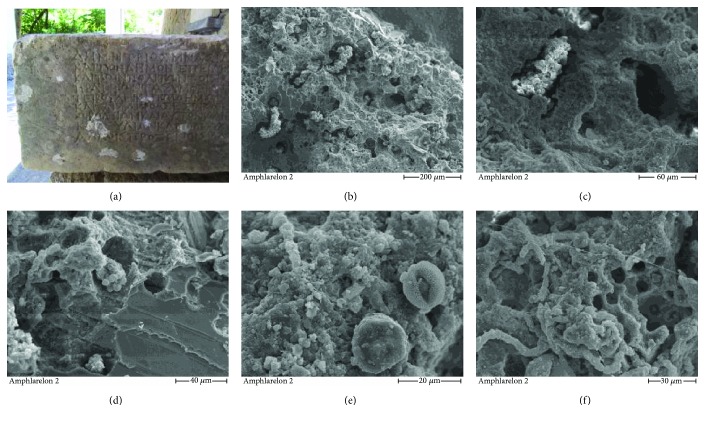
(a) The marble abacus under examination, Archaeological Site of Amphiareion in Oropos, Greece; SEM images of marble sample under examination showing (b, c) biopitting and microcolonial fungi (MCF) of the black yeast type; (d) biopitting and fresh breaking cleavage; (e) biofilm, pollen grain, and filaments of actinomycetes; and (f) lichen fungal mycelium, fungal hyphae, biofilm, and pitting.

The Archaeological Site of Amphiareion in Oropos, Greece, was an oracle dedicated to the ancient hero of Amphiaraos, built in the late 5th century BC. As many archaeological sites, Amphiareion presents extensive biodeterioration on its building elements. Samples from marble surfaces presenting discoloration and biological degradation were collected to be evaluated. The abacus depicted in Figure 6(a) was one of the stone elements under investigation and is a representative one regarding the preservation state of the Amphiareion marble surfaces. SEM investigation of a sample taken from a black-grey area of the marble abacus demonstrated the presence of microcolonial fungi (MCF) of the black yeast type. In particular, a large part of the marble surface presents biopitting by clearly evident MCF individual cells which penetrate deeply into the marble matrix (Figures 6(b) and 6(c)). Furthermore, in Figure 6(d), biopitting and fresh breaking cleavage that shows the disastrous speed of marble bioetching through the microcolonial fungi action are evident, while at the left part of the image, a filamentous growth starting a new microcolony made exclusively by yeast-like cells is observed. A pollen grain and filaments of actinomycetes attached to the biofilm are detected in Figure 6(e), whereas at the upper left of the image one large fungal filament covered by neomineral crystals is noticed. Finally, a lichen fungal mycelium, a fungal hyphae, biofilm, and pitting by individual cell clusters or filaments which penetrate vertically into the marble are presented in Figure 6(f). Therefore, despite the macroscopic initial impression of a discolored but firm external marble surface, it is demonstrated by SEM that both epilithic and endolithic lichens and fungi are present, disintegrating the marble matrix chemically and mechanically.

#### 2.2.2. Historical Mortar Technology

The examination of textural characteristics of historical mortars is, also, an important field regarding the materials science in built cultural heritage. Microtextural examination is achieved by SEM investigation and provides valuable information on the properties, the performance of historical mortars and concretes, the production technology [41], and the evolution of new phase formation [42]. Information about mortar morphology and texture regarding the interface cohesion of binder aggregates [43], as well as study of chemical composition, can be accomplished by SEM-EDX utilisation [44]. Lumps found within the mortar matrix [45], as well as reaction rims between pozzolanic additives and lime binders [46], are observed by SEM-EDX, shedding light to the production technology adopted. Furthermore, decay induced by salt crystallisation into mortars' pores can be also monitored [47].

The knowledge of the physicochemical and physicomechanical properties of historical mortars is the basis for the design of compatible restoration ones [48, 49]. SEM-EDX is a valuable technique throughout this design and decision-making procedure, as various studies in literature imply [43, 50].

In the study of the historical mortars of the Plaka Bridge in Epirus, Greece, SEM-EDX analysis provided important information about their microstructure characteristics and thus on their production technology. Plaka Bridge was built in 1866 and, because of its unique architecture, is considered a characteristic monument of the Epirus region. In 2015, its central part collapsed, and an interdisciplinary study for its restoration was undertaken by the National Technical University of Athens [51].

**Figure 7:**
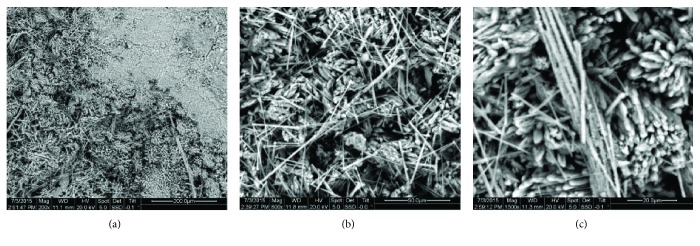
SEM images of a historical mortar from Plaka Bridge, Greece. (a) General view of the mortar binder; (b) and (c) fibrous particles aggregating to rods crossing the mortar matrix.

**Figure 8:**
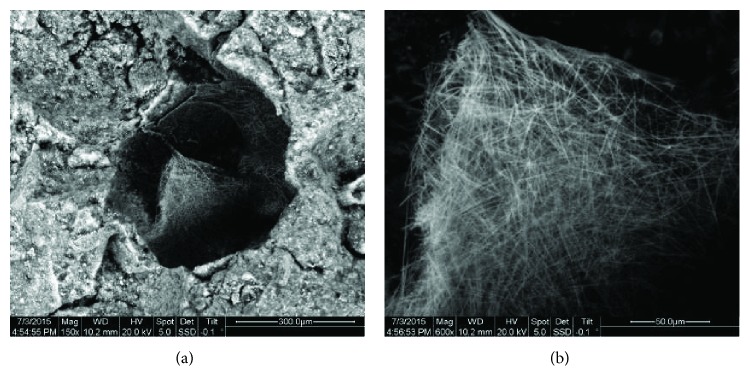
SEM images of a historical mortar from Plaka Bridge, Greece. (a) General view of the aggregate within the mortar matrix; (b) zoom in the mortar aggregate structure: fibers nestled in a mesh.

Historical mortars of Plaka Bridge were thoroughly investigated during this study, in order to design performing and compatible restoration mortars [52]. It was concluded that Plaka historical mortars are lime-pozzolan ones, presenting high hydraulicity [49]. Mercury intrusion porosimetry (MIP) measurements showed high porosity values (around 43% in average), high specific surface area values (19.47-49.49 m^2^/g), low average pore radius values (0.013-0.047 *μ*m), and low bulk density values (1.11-1.64 g/cm^3^) [49, 52]. These microstructure characteristics are attributed to the extended network of fibrous particles that cross the mortar binder and in many cases are shaping elongated rods, as well as to the use of fibrous minerals as aggregates (Figures 7 and 8, respectively).

It seems that, since the Plaka Bridge, as a one-arched bridge, had special construction requirements, these requirements included the use of a special traditional technology for mortar production: lightweight mortars with low average pore radius and such pore size distribution to hinder the capillary rise of the river water.

#### 2.2.3. Assessment of Conservation Materials and Treatments

The need for a solid analytical methodology in order to assess conservation interventions using certain performance criteria is pointed out in the literature [31, 53, 54]. Cleaning, consolidation, and protection are some of the most common interventions that take place in the field of architectural heritage. The choice of a conservation material/method is based on multiple parameters that mainly consider issues of compatibility between treatments and substrates; however, each application should be both laboratory- and pilot-tested on the scale of the historical building under investigation, to ensure that compatibility requirements are actually satisfied.

SEM-EDX is a powerful tool for the aforementioned aim, since diverse data regarding texture, morphology, and composition can be collected about the “composite” system of conservation material-historical substrate, for many aspects of conservation science such as (a) optimisation and performance assessment of consolidation treatments using nanolime materials [19], novel inorganic methods [55, 56], and hybrid nanomodified materials [57]; (b) assessment of water repellent performance regarding their penetration into porous stones [58]; and (c) assessment of cleaning treatments on stone crusts [59, 60], and on different types of graffiti [61–64].

One of the most important criteria regarding the cleaning assessment of black crusts is the chemical–mineralogical composition of the cleaned surface along with its stratification. SEM-EDX analysis of samples, taken from a black crust surface before and after cleaning, cut in cross sections, gives valuable information about the surface microstructure, the layers that have been retained and/or removed, the patina preservation (if it is present), and the possible cleaning by-products or other adverse effects that cleaning may have induced on stone.

**Figure 9:**
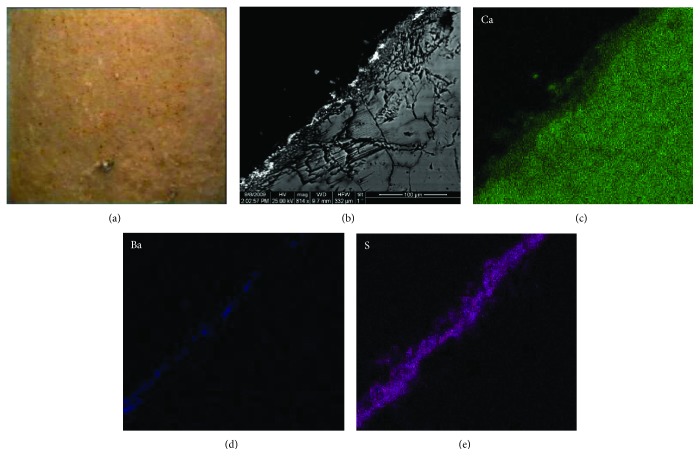
(a) Digital microscopy image of black crust before cleaning, capital, National Archaeological Museum of Athens, Greece (×25); (b) SEM BSE image of the under examination black crust (c, d, e) EDX mapping of the detected elements, Ca, Ba, and S, respectively.

Following the decay diagnosis of the NAM capitals and the patina identification, as presented in the previous section (2.2.1), pilot cleaning interventions were applied in situ and an assessment methodology was adopted, incorporating SEM-EDX among several other evaluation techniques [31]. One of the cleaning methods used for the removal of the black crust was an ion exchange resin with deionized water applied for 20 minutes.  Figure 9 presents the SEM-EDX results of the specific capital surface before cleaning. In this case, the patina layer presented only barite (BaSO_4_) and no zinc sulphide, which can be attributed to higher decay rates that zinc sulphide presents, in contrast to barite which is a rather stable and inert compound. Furthermore, the barite layer is located in the outer zone of a clear gypsum layer presenting microcrystalline texture.

**Figure 10:**
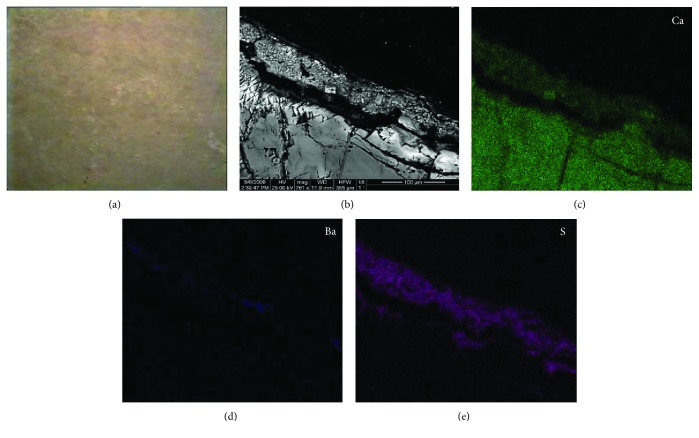
(a) Digital microscopy image of black crust after cleaning, using ion-exchange resin with deionized water for 20 min, capital, National Archaeological Museum of Athens, Greece (×25); (b) SEM BSE image of the under examination cleaned surface; (c, d, e) EDX mapping of the detected elements, Ca, Ba, and S, respectively.

Figure 10 presents SEM-EDX results after the application of the ion-exchange resin with deionized water for 20 minutes. According to the set cleaning assessment criteria [31], this cleaning method is classified as an accepted one, but it cannot be recommended for final application. Even though the microcrystalline gypsum layer is well-retained, the patina is not preserved at the required level, since the continuity and the thickness of the barite layer does not resemble the corresponding one before cleaning.

**Figure 11:**
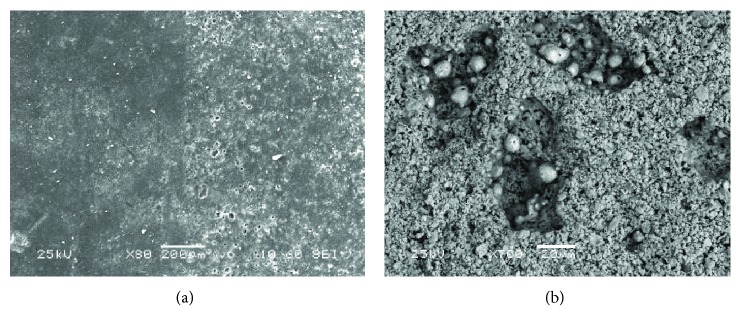
(a) SEM images of surface morphology for assessing a laser cleaning process of a dolomitic marble. Marble surfaces after laser cleaning at 1064 nm on the right part of the images and remains of graffiti covering surface on the left; (b) SEM images of surface morphology for assessing a laser cleaning process of a lead white painting. Lead white layer after laser cleaning at 1064 nm shows craters of different sizes with the formation of lead globule.

Furthermore, the morphological 3D-SEM images allow establishing the proper conditions for cleaning buildings stained by graphites or deposits and monitoring the weathering forms [61].  Figure 11(a) shows the monitoring of dolomitic marbles cleaned with a Nd:YAG laser at 1064 nm. In this stone, widely employed in buildings, pores are mainly intercrystalline due to grain contacts and no structural damages were observed after laser cleaning, and a surface morphology and porosity similar to fresh marble is observed. On the other hand, SEM can evidence the damage on painting layers on a cleaning process with Nd:YAG laser at 1064 nm.  Figure 11(b) shows a lead white layer after laser cleaning at 1064 nm and evidence the crater's occurrence of different sizes with the formation of lead globule. Because of that, this cleaning technique with Nd:YAG laser at 1064 nm cannot be recommended for painting walls where lead white could be employed as pigment or preparation layer.

**Figure 12:**
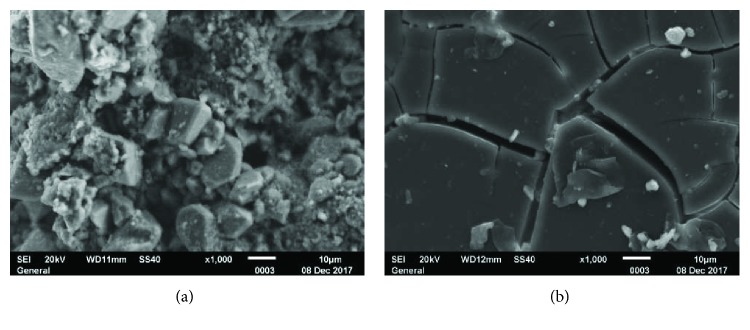
(a) Treatment of nanolime on limestone from Puerto de Santa María (Cádiz, España). Ca(OH)_2_ nanoparticles enter in stone and consolidate the grains. (b) Layer of silica nanoparticles over the surface of limestone from Espera (Cádiz, España), showing the incompatibility of the treatment with this kind of limestone.

The development of new nanoconsolidants based on nanolimes (Ca(OH)_2_ nanoparticles) or silica nanoparticles (SiO_2_ nanoparticles) is widely studied by SEM since a morphological point of view in order to evaluate the compatibility with the stone substrate.  Figure 12(a) shows limestone coming from the quarry of Puerto Santa Maria (Cadiz, Spain) with a treatment based on nanolimes where Ca(OH)_2_ nanoparticles are occupying intercrystalline porosity, consolidating the carbonated and silica grains with calcite, while Figure 12(b) shows a silica nanoparticle treatment, where a layer of silica is observed on the surface and does not cross the surface of the limestone from Espera (Cadiz, Spain). SEM allows us to explain the incompatibility of silica nanoparticles with this limestone, and therefore, the former treatment based on nanosilica cannot be recommended for this kind of limestone.

## 3. TEM Analysis

### 3.1. Instrumentation and Sample Preparation

Like SEM, TEM consists of four components: an electron optical column, a vacuum system, electronics (lens supplies for focusing and deflecting the beam and the high voltage generator), and control software. The electron beam emerges from the electron gun (at the top of the column) and is condensed into a nearly parallel beam at the specimen by the condenser lenses. After passing through the sample, transmitted electrons are collected and focused by the objective lens and a magnified real image of the sample is projected by the projection lenses onto the viewing device at the bottom of the column.

The most important differences between SEM and TEM are as follows:

- TEM uses a broad static beam while SEM n beam is focused to a fine point and scanned line by line the sample surface in a rectangular raster pattern
- The accelerating voltage in TEM is much higher since penetration of the specimen is required
- A more sophisticated sample preparation; it is important to make the specimen stable and small enough (some 3 mm in diameter) to permit its introduction into the evacuated microscope column and thin enough to permit the transmission of electrons

Regarding sample preparation for TEM analysis, each scientific branch has its own sample thickness requirements, but the specimen must be thin enough to transmit the electrons, typically 0.5 *μ*m or less.

The TEM mode that is commonly used in the field of cultural heritage is the bright-field (BF) imaging method. In this case, areas including elements of higher atomic numbers are presented darker, compared with the ones that contain lighter elements [65].

### 3.2. TEM Analysis in Built Cultural Heritage

TEM applications in cultural heritage involve mostly archaeometric studies of relatively homogeneous materials. Nanocrystals can be effectively observed in the amorphous matrix of several materials such as ceramics, glaze, and glass [66]. In building materials research, the technique has been employed for the evaluation of the performance of consolidation treatments [19], as well as the examination of the microstructure of various pozzolanic mortars [67, 68].

**Figure 13:**
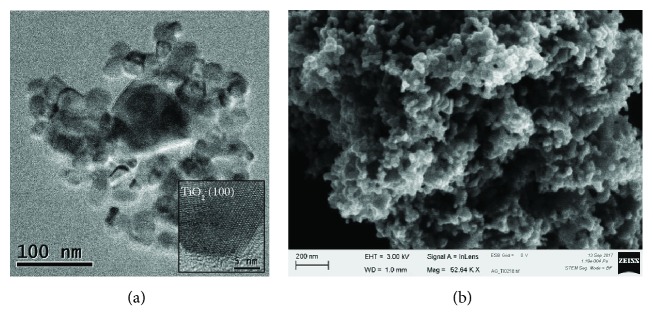
(a) TEM image of a nanocomposite based on silver and TiO_2_ nanoparticles. Inset, identification of the mineral phase of TiO_2_ nanoparticles as anatase; (b) SEM image of an aggregation of silver and titanium dioxide nanoparticles.

The combination of TEM and SEM is very useful for the characterization of new nanocomposites, as TEM allows identifying mineral phases and the relationship between the nanoparticles that constitute a nanocomposite, and SEM informs about the morphology of the nanocomposite and their trend to aggregate. This last factor must be considered before the application of the treatment on stone [69].  Figure 13 shows an example of the combined use of TEM and SEM to study a nanocomposite based on silver and titanium dioxide nanoparticles. The interaction between silver and TiO_2_ nanoparticles was studied by TEM. Additionally, as can be seen in the image inset in Figure 13(a), the high-resolution TEM image allowed identifying the TiO_2_ nanoparticles used in the nanocomposite as anatase, measuring the spacing of the lattice fringes which corresponded to the [1 0 0] lattice plane of the anatase. The SEM image of the same nanocomposite (Figure 13(b)) provides tomographic information to identify the morphological characteristics of the different nanoparticles used in this nanocomposite (shape and size) and their trend to aggregate in the absence of solvent.

Among recent publications, we introduce here the use of SEM and TEM for the morphological characterization of antifouling nanoparticles for heritage application [70]. Based on recent and stricter regulations, many commercial biocides were gradually banned for toxicity. The use and application of an antifoulant in heritage preservation, in particular for archaeological area and outdoor artworks and buildings, is of crucial importance considering critical aspects such as efficiency, sustainability, and toxicity for both the operators and the surrounding environments. The work showed the synthesis and characterization of silica nanocontainers loaded with zosteric sodium salt, a natural product antifoulant (NPA).

**Figure 14:**
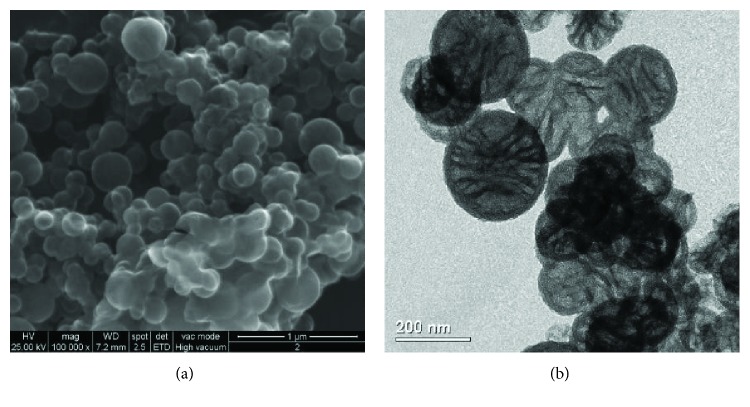
(a) SEM and (b) TEM images of the starting empty nanocapsules (NC) (reproduced with authorisation from [70]).

**Figure 15:**
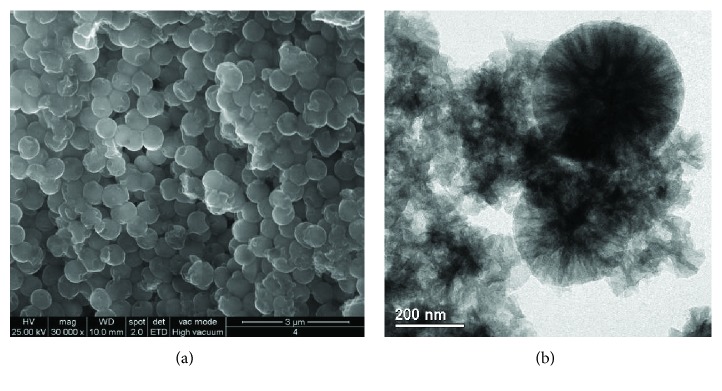
(a) SEM and (b) TEM images of the silica nanocapsules loaded with zosteric sodium salt (ZS) (reproduced with authorisation from [70]).

Two different compounds, sodium benzoate (BS) and zosteric sodium salt (SZ), were tested and encapsulated in silica nanoparticles. Figures 14(a) and 14(b) show typical SEM and TEM images of empty silica nanocapsules (NC). The TEM image (Figure 15(b)) depicts the spherical shape of the nanoparticles and shows their hollow nature, as highlighted by the contrast between the dark edge and the centre.

**Figure 16:**
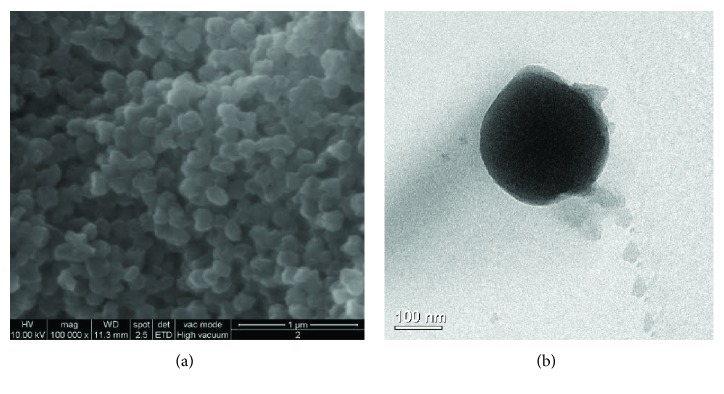
(a) SEM and (b) TEM images of the nanocontainers loaded with BS (reproduced with authorisation from [70]).

The comparison of the SEM and TEM images of the NC with the nanocapsules loaded with the synthesised biocides was needed to verify that the process did not promote any variations in terms of shape, but in size only. Based on the TEM-associated size distribution (shown in [70]), the size of the nanocapsules (NC) is centered at 148 nm, while the distribution of the containers loaded with ZS and BS is at 563 and at 164 nm, respectively. Figures 16(a) and 16(b) show the SEM and TEM images of the ZS- and BS-encapsulated particles, respectively.

The images highlight, in particular, how both samples, compared to the starting nanosilica containers, appear agglomerated probably due to the presence of organic residual. The application of SEM and TEM analytical techniques is in this case fundamental not only for the validation of the encapsulation process but also for understanding the nanocontainers' interaction with the products. In the case of biocides, the presence of the products within the containers is a crucial aspect in order to assure a controlled release of nontoxic and environmentally friendly biocides.

TEM measurements have also proved extremely useful in the examination of the interface and possible reaction rims between the binder part and the aggregates of historical mortars, thus revealing their production technology and explaining their excellent properties.

Pozzolanic mortars can be found in several archaeological sites and monuments, as early on as 1500 BC (e.g., wall covering in Santorini Island, Greece). In the Byzantine and Ottoman period, many monuments were constructed with the use of lime mortars with crushed brick, where the crushed brick plays the role of an artificial pozzolan. These monuments have shown excellent behaviour throughout the ages and despite negative environmental factors.

**Figure 17:**
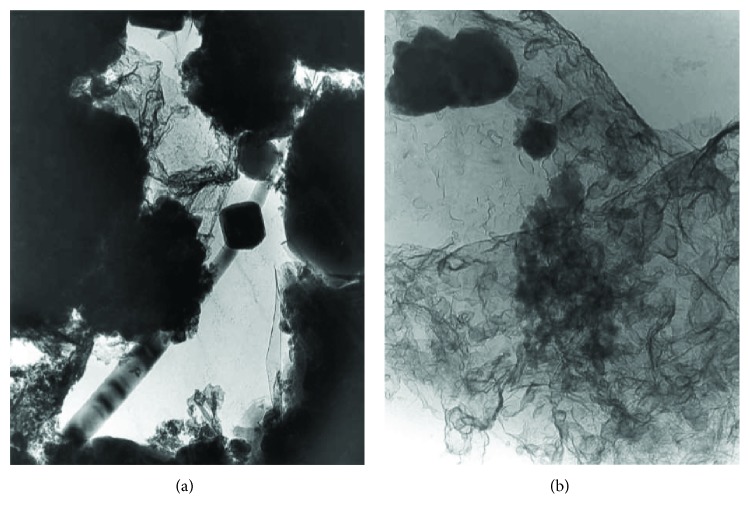
TEM results of Hagia Sophia mortars: (a) amorphous C-S-H gel formation developed between crystalline phases of calcite and the dispersed ceramic fragments and quartz crystals in a mortar sample from Hagia Sophia, magnification: ×22,000; (b) the C-S-H gel presents a sheet structure, while in certain areas (lower left of image) quasi-crystalline phases are discerned, in the dome rib mortar sample from Hagia Sophia, magnification: ×42,000.

Specifically, in Figure 17, TEM measurements revealed the presence of a C-S-H gel crystalline interface, formed between the calcitic matrix and the exterior of the brick aggregates of the Hagia Sophia mortars [71]. This phase is similar to the C-S-H gel phases which are formed in cement mortars and explains the durability of the Hagia Sophia throughout the centuries, as well as its excellent response under earthquake stresses, as these amorphous hydraulic formations (CSH), apart from imparting higher mechanical strength values to the mortar, also allow for greater energy absorption without initiations of fractures.

**Figure 18:**
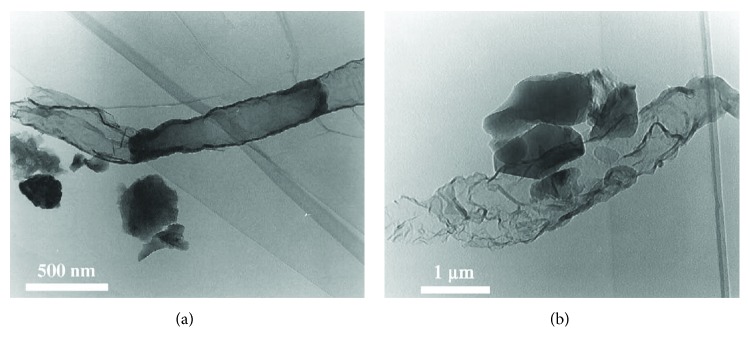
(a) TEM image of the filament-like entities, which are recognized as a C-S-H phase, formed in a traditional pozzolanic mortar from Rhodes; (b) TEM image revealing the foil-like morphology of the C-S-H phase, product of the pozzolanic reaction, in a traditional pozzolanic mortar from Rhodes.

The same gel, with a foil-like morphology, was also noticed in historical traditional brick mortars found on various monuments in the island of Rhodes (Figure 18), further affirming its positive effect and the widespread use of this mortar production technology [67]. These findings provided the basis for the design of appropriate restoration mortars which could exhibit these supreme qualities in addition to enhanced compatibility, and TEM can serve as a tool in confirming the optimum formation of the C-S-H gel.

## 4. Conclusions

The most widely used scanning microscopy techniques in the field of the protection of built cultural heritage are scanning electron microscopy and the transmission electron microscopy.

SEM-EDX applications are widespread and commonly found in the field of built heritage, and therefore, SEM-EDX can be considered a routine technique, rather than a research technique, for (a) building material characterization, (b) patina identification, (c) environmental impact assessment on building materials regarding pollution, salt crystallisation, and biodeterioration, and (d) assessment of conservation materials and treatments, such as cleaning, consolidation, and protection. The morphological, microstructural, and chemical information that can be collected and recorded by a SEM-EDX application, in combination with the little to no need of sample preparation, has established the key role of this technique in the field of built cultural heritage, where sampling is rather limited, and therefore techniques which allow approaches of diverse data are significantly valuable.

TEM applications, on the other hand, are not so commonly found in the field of built heritage, mainly due to the heterogeneity that building materials present, a fact that leads to reduced applicability of this technique, without ignoring the demanding procedures of sample preparation, especially when considering that sample preparation has a great impact on the quality of TEM results. However, TEM becomes an indispensable tool in cases such as (a) the detection and examination of nanostructures in historical building materials, disclosure of their production technology and interpretation of their properties, and (b) the development evaluation of cutting-edge nanomaterials, specially designed for the conservation of built heritage.

Technological advances will further evolve the capabilities and the applicability not only of the two aforementioned techniques but also of other scanning microscopy techniques, in material science, contributing to the application of improved methodological approaches for the sustainable protection of built cultural heritage.

## Figures and Tables

**Table 1:** Summary of analytical techniques used in the field of built cultural heritage, regarding acquired information, sample requirements, and major limitations.

Techniques	Acquired information	Sample requirements	Limitations
Atomic absorption/emission spectroscopy AAS/AES	(i) Elemental chemical composition (ii) High sensibility & precision for quantitative analysis (ppm/ppb)	(i) Solid samples are converted to liquid solutions	(i) Time-consuming sample preparation (ii) Sample destruction
Inductively coupled plasma optical emission spectrometry ICP-OES			
Fourier transform infrared spectroscopy FTIR	(i) Molecular chemical composition (ii) Qualitative analysis	(i) Small amount of solid & liquid samples (ii) No sample preparation for ATR-FTIR mode (iii) Nondestructive in situ instrumentation	(i) Peak overlay and/or shifting, when mixtures are measured (ii) Database is required
RAMAN spectroscopy	(i) Molecular chemical composition (ii) Qualitative analysis	(i) No preparation of solid samples (ii) Nondestructive in situ instrumentation	
X-ray fluorescence XRF	(i) Elemental chemical composition (ii) Quantitative analysis	(i) No preparation of solid samples (ii) Nondestructive in situ instrumentation	(i) Quantification uncertainty (ii) Elements of low atomic number are not detected
X-ray diffraction XRD	(i) Mineralogical composition (ii) Semiquantitative analysis	(i) Pulverising of solid samples (ii) Nondestructive in situ instrumentation	(i) Detection limit above ~5% (ii) Database is required
Particle-induced X-ray emission/particle-induced gamma-ray emission PIXE/PIGE	(i) Elemental chemical composition	(i) No preparation of solid samples	(i) Quantification uncertainty (ii) Elements of low atomic number are not detected
Mass spectrometry MS	(i) Isotopic determination	(i) Pulverising of solid samples	(i) Risk of sample contamination
Gas chromatography/high-performance liquid chromatography/ionic chromatography GC/HPLC/IC	(i) Separation techniques (ii) GC/HPLC mainly employed in organic compounds (iii) IC employed in inorganic compounds	(i) Samples must be solubilised	(i) Time-consuming sample preparation (ii) Requirement of internal and external standards
Laser-induced breakdown spectroscopy LIBS	(i) Elemental chemical composition (ii) Qualitative & quantitative analysis	(i) No sample preparation (ii) In situ instrumentation	(i) Surface destructive technique (ii) Database is required
Laser-induced fluorescence LIF	(i) Molecular chemical composition	(i) No sample preparation (ii) In situ instrumentation	(i) Database is required (ii) Only fluorescence compounds are detected

**Table 2:** Summary of the main advantages and limitations of SEM, TEM, and AFM in the field of build cultural heritage.

Technique	Advantages	Limitations
Scanning electron microscopy, SEM	(i) No sample treatment (ESEM), or simple application of gold and/or carbon coating (ii) Quantitative analysis when coupled with EDX	(i) Certain working conditions can alter sample surface (ii) Small area examination is not always representative
Transmission electron microscopy, TEM	(i) Nanoscale resolution (ii) Quantitative analysis when coupled with EDX	(i) Sophisticated sample treatment is required (ii) Sample treatment can affect quality of results (iii) Small area examination is not always representative
Atomic force microscopy, AFM	(i) No sample treatment is required (ii) Nanoscale resolution (iii) Acquisition of 3D images	(i) Not applicable when surface texture is above nanoscale (ii) Small area examination is not always representative
